# Linkage to Care and Treatment for TB and HIV among People Newly Diagnosed with TB or HIV-Associated TB at a Large, Inner City South African Hospital

**DOI:** 10.1371/journal.pone.0049140

**Published:** 2013-01-16

**Authors:** Yara Voss De Lima, Denise Evans, Liesl Page-Shipp, Antonia Barnard, Ian Sanne, Colin N. Menezes, Annelies Van Rie

**Affiliations:** 1 Clinical HIV Research Unit, Faculty of Health Sciences, University of the Witwatersrand, Johannesburg, South Africa; 2 Health Economics and Epidemiology Research Office, University of the Witwatersrand, Johannesburg, South Africa; 3 Right to Care, Johannesburg, South Africa; 4 Gauteng Department of Health and Social Development, Johannesburg, South Africa; 5 Infectious Diseases Unit, Department of Medicine, Faculty of Health Sciences, University of the Witwatersrand, Johannesburg, South Africa; 6 Department of Epidemiology, University of North Carolina at Chapel Hill, Chapel Hill, North Carolina, United States of America; Vanderbilt University, United States of America

## Abstract

**Objective:**

To assess the outcomes of linkage to TB and HIV care and identify risk factors for poor referral outcomes.

**Design:**

Cohort study of TB patients diagnosed at an urban hospital.

**Methods:**

Linkage to care was determined by review of clinic files, national death register, and telephone contact, and classified as linked to care, delayed linkage to care (>7 days for TB treatment, >30 days for HIV care), or failed linkage to care. We performed log-binomial regression to identify patient and referral characteristics associated with poor referral outcomes.

**Results:**

Among 593 TB patients, 23% failed linkage to TB treatment and 30.3% of the 77.0% who linked to care arrived late. Among 486 (86.9%) HIV-infected TB patients, 38.3% failed linkage to HIV care, and 32% of the 61.7% who linked to care presented late. One in six HIV-infected patients failed linkage to both TB and HIV care. Only 20.2% of HIV-infected patients were referred to a single clinic for integrated care. A referral letter was present in 90.3%, but only 23.7% included HIV status and 18.8% CD4 cell count. Lack of education (RR 1.85) and low CD4 count (CD4≤50 vs. >250cells/mm^3^; RR 1.66) were associated with failed linkage to TB care. Risk factors for failed linkage to HIV care were antiretroviral-naïve status (RR 1.29), and absence of referral letter with HIV or CD4 cell count (RR1.23).

**Conclusions:**

Linkage to TB/HIV care should be strengthened by communication of HIV and CD4 results, ART initiation during hospitalization and TB/HIV integration at primary care.

## Introduction

South Africa is one of the 22 countries with a high tuberculosis (TB) burden [1]. In 2009, almost half a million new cases of tuberculosis were notified in South Africa, [2] corresponding to a rate of 971 cases per 100,000 population. The burden of HIV-associated tuberculosis in South Africa is enormous, with an estimated 73% of TB cases being HIV-infected, and one in four of all global HIV-associated TB cases occurring in South Africa [1].

Adherence to treatment for TB and HIV is an important determinant of a favourable treatment outcome, and those who default treatment, especially if soon after treatment initiation, continue to be a source of infection and transmission within the community [3], [4]. Patients with drug resistant TB that default therapy pose an even greater risk to the public [5], [6]. It is estimated that a person with smear-positive TB will infect 10–14 people per year [7]. The risk of transmitting HIV is mainly related to the HIV viral load and level of risk behaviour [8]. While treatment interruption during the course of TB treatment and antiretroviral treatment (ART) have been extensively studied [9]–[13], little is known about the linkage to care after the initial diagnosis [14], particularly among people diagnosed with HIV-associated TB at a hospital facility [15], [16]. Because the cost-effectiveness of diagnostic interventions is in part determined by successful linkage to care [17], [18], the rate of linkage to care should be monitored and evaluated along with other programmatic targets [18], [19]. Currently, rates of and reasons for failure to link TB and HIV diagnosis with care are not collected as part of routine monitoring and evaluation in TB and ART programs, making evidence-based decisions on how to improve linkage to care difficult [20].

We aimed to determine the proportion of people who fail to link to care for TB and HIV treatment following a TB diagnosis at an inner city hospital in Johannesburg, South Africa, and assess risk factors for delayed or failed linkage to TB and HIV care.

## Methods

### Ethics statement

Study approval was obtained from the Human Research Ethic Committee of the University of Witwatersrand, Institutional Review Board of the University of North Carolina, the City of Johannesburg (CoJ) municipality and the Helen Joseph hospital. A waiver of written informed consent was granted by the ethics committees (retrospective review of existing medical records). Verbal informed consent for use of information provided by individuals contacted by phone was obtained from all individuals and documented in writing on the informed consent form used. All data was de-identified prior to analysis and analysed anonymously.

### Setting and patient population

The TB focal point (TBFP) at the Helen Joseph public hospital in Johannesburg, South Africa, provides routine TB diagnosis for inpatients and some outpatients, TB education, and HIV counselling, testing and staging for TB suspects and TB patients. All activities are performed according to National TB guidelines [21], [22]. During the study period, the South African guidelines recommended ART initiation as soon as the patient is stable and completed at least two weeks of TB treatment if CD4 count is less than 50 cells/mm or in the presence of other serious HIV “illnesses”; at the end of the two-month intensive phase of TB therapy if CD4 count is between 50 cells/mm and 200 cells/mm; and after completion of TB therapy if CD4 count was above 200 cells/mm and there were no other HIV-related symptoms.

Patients are referred for continuation of TB treatment to the most appropriate local primary health care clinic based on the patient's home or work address, and provided with TB medication for 7 days. Patients on ART are provided with antiretroviral drugs for 30 days and ART-naïve HIV-infected patients are instructed to present for HIV care and treatment within 30 days of referral. National guidelines state that if possible, the patient should either be transported to the clinic by health services, be accompanied by a DOT supporter or social worker, or the local PHC clinic should collect the patient. If this is not possible the hospital should follow up directly with the clinic to confirm that the patient has arrived [21], [22]. Because the TBFP does not have the capacity to accompany patients or to confirm arrival of all patients, a standard South African TB Programme referral letter (“pink”) form is given to the patient to deliver to the referral clinic. A copy of this referral letter is sent directly to the clinic where the patient is referred to, and a third copy is kept at the TBFP. The guidelines further instruct that the pink referral form must be completed in detail with all relevant information. [21], [22] While the form includes a field “other information”, there is no dedicated space for HIV status or CD4 count.

All adults (age 18 years or older) registered with active TB at the TBFP with TB between October 1 and December 31, 2009 and referred to a clinic within the CoJ health district were included in the analysis.

### Data collection

The TBFP routinely collects socio-demographic data, clinical information, laboratory results related to TB and HIV care, patient contact information and the name of the clinic(s) patients are referred to for TB and HIV treatment.

**Figure 1:**
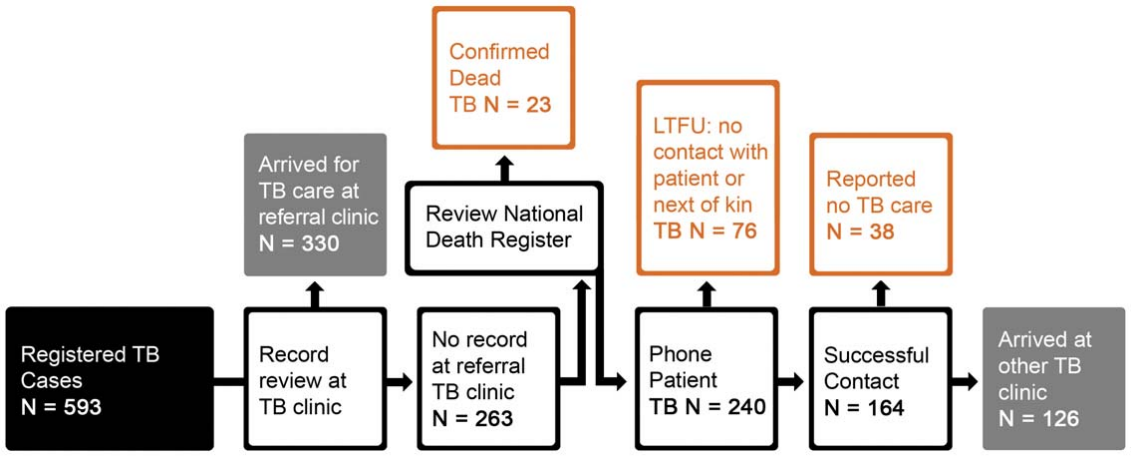
Flow chart representing the assessment of linkage to TB treatment. This flow chart details the data collection and verification process used to determine the outcome of referral of 593 TB patients for continuation of TB treatment at primary care level. Data was collected three months (median 192 days) after referral to ensure capture of data on individuals who presented late after referral.

**Figure 2:**
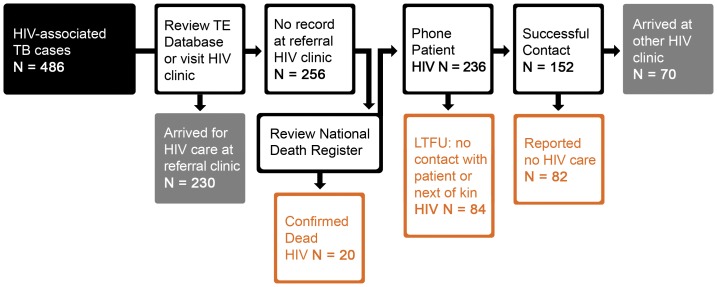
Flow chart representing the assessment of linkage to HIV care and treatment. This flow chart details the data collection and verification process used to determine the outcome of referral of 486 HIV co-infected patients with TB for continuation of HIV care and treatment. Data was collected 3 months (median 192 days) after referral to ensure capture of data on individuals who presented late after referral.

For the study, data on the outcome of the referral process was collected 3 months (median 192 days, range 161–231) after referral from TBFP to ensure capture of late presenters (Figures 1 and 2). Referral clinics were visited to confirm patient arrival by interviewing the TB nurse and reviewing the clinic TB register. The patient TB file was reviewed to determine the exact date of patient arrival, standard referral form for documentation of HIV and CD4 cell count information, locate the pink form or any other referral documents, and review documentation on HIV status, care and treatment. For patients with HIV-associated TB, TherapyEdge™ an electronic database, used by several HIV clinics in Johannesburg, was reviewed to establish linkage to HIV care. CoJ clinics that did not use Therapy Edge-HIV™ were visited. In total, study staff visited 98 clinics within CoJ.

When documentation of patient arrival at the referral clinic for TB and HIV treatment was not found, the electronic South Africa's National Vital Registration system was consulted to verify the alive or deceased status of the patient. Patients that were alive or with unknown survival status were contacted telephonically using a standardized script to enquire about TB and, if applicable, HIV treatment. If patients reported they had presented for TB and/or HIV treatment, the name of the clinic was recorded and the clinic was visited to confirm attendance.

### Data analysis

The primary outcome was time to arrival at the referral clinic, categorized as (i) linked to care, (ii) delayed linkage to care (iii) failed of linkage to care. Delayed linkage to care was defined as presentation for TB treatment more than 7 days or presentation for HIV care and treatment more than 30 days after hospital discharge, corresponding to the 7 day supply of TB treatment, 30 day supply of antiretroviral drugs, or a 30 days period during which the ART-naive patients were asked to present for HIV care. Failed linkage to care included patients who self-reported not being in care as well as patients who had no record of care and could not be traced by phone, assuming they had not presented for care. Initiation of care in people who could not be traced could not be excluded. We therefore explored the potential of misclassification in a sensitivity analysis in which all patients who could not be traced were classified as having linked to care. Patients who died within 7 days or 30 days of referral for TB or HIV care, respectively, were classified as deceased during referral process.

Multiple log-binomial regression analysis was performed to identify patient and referral characteristics independently associated with delayed and failed linkage to TB and HIV care, respectively. Measures of association were expressed as relative risks (RR) and their 95% confidence intervals (CI). Variables associated with the outcome in bivariate analysis at p≤0.20 were considered in regression models. Data was analyzed using SAS 9.1 statistical software package (SAS institute, Inc., North Carolina, USA.

## Results

### Patients characteristics

During the 3-month study period, 678 adults were registered with active TB at the TBFP, of which 593 were referred to a CoJ clinic and included in the analysis. Demographic and clinical characteristics of patients referred to CoJ clinics did not differ from the overall cohort (p>0.2). Median age was 35.9 years (IQR 30.6–43.5), 50% were female, 82.6% were of South African nationality, 80% had an education level of grade 8 or higher, and 54.7% were unemployed (Table 1). The majority (68.5%) had smear-negative TB. Most patient (86.9%) were HIV-infected, 8.9% were HIV negative and 4.2% of patients had an unknown HIV status. Median CD4 cell count at TB diagnosis was 78 cells/mm^3^ (IQR 30–178). At time of referral, only 21% of HIV-infected patients were receiving ART.

**Table 1:** Baseline demographics and clinical characteristics of 593 patients diagnosed with TB at the Helen Joseph Hospital TB focal point.

Patient characteristic		N or median	% or IQR
Gender (n, %)	Male	295	50
	Female	298	50
Age at TB-FP (years)	Median (IQR)	35.9	30.6–43.5
	≤40	395	66.6
	>40	198	33.4
Nationality	South African	490	82.6
	Zimbabwe	93	15.7
	Other	10	1.7
Time in Johannesburg <6 m	No	454	92.8
	Yes	35	7.2
Employment	No	321	54.7
	Yes	265	45.3
Education	No education	30	5.1
	<Grade 8	88	14.9
	≥Grade 8	471	80.0
HIV status	HIV negative	53	8.9
	HIV positive	515	86.9
	On ART	108	21.0
	Not on ART	407	79.0
	Unknown status	25	4.2
CD4 cell count	Median (IQR)	78	30–178
	≤50 cell/mm^3^	186	37.3
	51–100 cell/mm^3^	101	20.3
	101–250 cell/mm^3^	138	27.7
	>250 cell/mm^3^	73	14.7

Time in Johannesburg was missing in 104 patients, employment status in 7 patients, education status in 4 patients for, and CD4 cell count results in 17 patients.

### Linkage to TB care

Overall, 137 (23%) patients failed to link to TB care and 456 (77%) presented to a clinic for continuation of TB care and treatment (Table 2). Among the 456 who had linked to care, 126 (27.6%) had presented to a clinic other than the one they had been referred to by TBFP staff. The median time to presentation was 5 days (IQR 2 -10) but 138 (30.3%) arrived late, after their 7 day supply of TB drugs expired. Among the 415 patients who arrived for TB care at a CoJ clinic, 375 (90.3%) had a clinic file that contained a referral letter but this letter contained the HIV status in only 23.7% of patients, and the CD4 cell count was communicated in as few as 18.8% of HIV-infected TB patients. Among the 137 (23%) patients classified as failure to link to TB care, 6 (4.4%) had died within the first 7 days after TBFP referral, 38 (27.7%) self-reported they had never presented at a TB clinic and 93 (67.9%) could not be traced.

**Table 2:** Outcome of referral from Helen Joseph hospital TB focal point to City of Johannesburg clinic for continuation of TB treatment (n = 593) and HIV care and treatment (n = 486).

Outcome of referral		N	% or IQR
**Linkage to TB care**	Arrived at TB clinic	456	77.0
	Arrived at original referral clinic	330	72.4
	Arrived at other clinic within COJ	85	18.6
	Arrival at TB clinic outside COJ	41	9.0
	Failed to link to TB care	137	23
	Patient confirms not attending any TB clinic	38	27.7
	Died within 7 days of discharge	6	4.4
	Loss to follow-up	93	67.9
Timing of linkage to TB care*	Median, IQR (days)	5	2–10
	Arrived on time (≤7 days)	277	60.7
	Arrived late (>7 days)	138	30.3
	Arrived (exact date missing)	41	9.0
Referral information at TB clinic**	Referral letter present in clinic file	375/415	90.3
	Referral letter with HIV status	89/375	23.7
	Among HIV positive, referral letter with CD4 count	68/361	18.8
	No discharge letter received at clinic	40/415	9.6
**Linkage to HIV care**	Arrival at HIV clinic	300	61.7
	Arrived at original HIV referral clinic	230	76.7
	Arrived at other HIV clinic within COJ	66	22.0
	Arrival at HIV clinic outside COJ	4	1.3
	Failed to arrive	186	38.3
	Patient confirms not attending any HIV clinic	82	44.1
	Loss to follow-up	94	50.5
	Died within 30 days of referral	10	5.4
Timing of linkage to HIV care***	Median, IQR (days)	17	5–48
	Arrived in time (≤30 days)	191	63.7
	Arrived late (>30 days)	96	32.0
	Arrived (exact date missing)	13	4.3
Type of referral clinic	Same TB and HIV referral site	98	20.2
	Two different referral sites for HIV and TB	386	79.8
Appropriateness of referral HIV clinic	Appropriate	426	87.7
	Not appropriate*	60	12.3

* Among 456 patients who linked to TB care;

** Among 415 patients who linked to TB care at a COJ clinic;

*** Among 300 HIV infected TB patients who linked to HIV care *Not appropriate = referral of an ART-naïve patients to clinic where ART is not initiated; CoJ = City of Johannesburg; IQR = interquartile range.

### Linkage to HIV care and treatment

Among the 515 patients with HIV-associated TB, 486 were referred to a CoJ clinic and included in the analysis. Of the 486 patients, 186 (38.3%) failed to link to HIV care and 300 (61.7%) presented for HIV care and treatment (Table 2). Among those that linked to HIV care, 66 (22.0%) had presented to a clinic different than the one they had been referred to by the TBFP staff. The median time to presentation was 17 days (IQR 5–48), but 32% presented late, more than 30 days after referral by the TBFP. Among the 186 patients classified as failed linkage to care, 10 (5.4%) had died within the first 30 days of referral, 82 (44.1%) admitted they had never presented at a HIV clinic, and 94 (50.5%) could not be traced.

The majority of patients (79.8%) had been referred to two separate clinics, one for TB treatment and one for HIV care and treatment. Among ART-naïve patients, 12% were inappropriately referred to a clinic not accredited for ART initiation.

### Correlation of linkage to TB and HIV care and treatment (in patients with recorded arrival times)

Among patients with HIV-associated TB, exact dates of arrival were available for 461 (91.5%) patients. One in six HIV-infected TB patients (16.7%) failed to link to both HIV and TB care and treatment, about half (55.5%) linked to both HIV and TB care and treatment; one in five (22.3%) linked to TB treatment but failed to link to HIV care, and few (5.4%) linked to HIV care and treatment but failed to link to TB treatment. Patients who failed to link to HIV care were also at increased risk of failure to link to TB care (RR 1.59, 95% CI 1.42–1.76).

### Factors associated with delayed or failed linkage to TB or HIV care and treatment

In bivariate analysis, being a non-South African (RR 1.42; 95% CI 1.04–1.92), lack of formal education (RR 2.06; 95% CI 1.36–3.11), and low CD4 (≤50 vs. >250 cells/mm^3^; RR 2.22; 95% CI 1.24–3.96) were associated with increased risk of failed linkage to TB care (Table 3). Risk factors for delayed linkage to care were being a non-South African (RR 1.38 95% CI 1.02–1.86), lack of formal education (RR 2.02, 95% CI 1.38–2.96), being ART-naïve at referral (RR 1.67; 95% CI 1.07–2.60), and the absence of a referral letter in the TB clinic file (RR 1.82, 95% CI 1.34–2.49). In multivariate analysis, the lack of formal education (aRR 2.12 95% CI 1.44–3.09) and absence of a discharge letter (aRR 1.47 95% CI 1.02–2.11) were independently associated with increased risk of delayed linkage to TB care. Lack of formal education (aRR 1.85 95% CI 1.31–2.61) and low CD4 (≤50 vs. >250cells/mm^3^; aRR 1.66 95% CI 1.07–2.64) were independently associated with increased risk of failed linkage to TB care.

**Table 3:** Factors associated with delayed or failed linkage to TB care in 593 patients referred from Helen Joseph hospital TB focal point to City of Johannesburg (COJ) clinic for TB treatment.

		Delayed linkage to care (>7 days) (n = 138)			Failed linkage to care (n = 131^#^)		
		N	Crude RR (95% CI)	Adjusted RR (95% CI)	N	Crude RR (95% CI)	Adjusted RR (95% CI)
Sex	Female	73	Reference		63	Reference	
	Male	65	0.94 (0.71–1.23)		68	1.07 (0.81–1.42)	
Age	<36 years	74	Reference		64	Reference	
	≥36 years	64	0.92 (0.70–1.21)		67	1.04 (0.80–1.39)	
Nationality	South African	103	Reference	Reference	97	Reference	Reference
	Other	35	1.38 (1.02–1.86)	1.39 (0.92–2.17)	34	1.42 (1.04–1.92)	1.18 (0.87–1.59)
Education	≥Grade 8	108	Reference	Reference	100	Reference	Reference
	<Grade 8	18	0.97 (0.64–1.46)	0.95 (0.62–1.46)	20	1.09 (0.74–1.63)	1.06 (0.75–1.49)
	No education	11	2.02 (1.38–2.96)	2.12 (1.44–3.13)	10	2.06 (1.36–3.11)	1.85 (1.31–2.61)
Unemployed	No	66	Reference		53	Reference	
	Yes	71	0.96 (0.73–1.26)		75	1.16 (0.87–1.55)	
ART status	On ART	17	Reference	Reference	22	Reference	
	ART-naive	110	1.67 (1.07–2.60)	1.39 (0.92–2.17)	91	1.22 (0.82–1.81)	
CD4 count	>250 cell/mm^3^	22	Reference	Reference	10	Reference	Reference
	101–250 cell/mm^3^	27	0.72 (0.46–1.15)	0.80 (0.51–1.25)	27	1.27.(0.67–2.40)	1.16 (0.71–1.83)
	51–100 cell/mm^3^	26	0.91 (0.36–1.43)	0.98 (0.65–1.49)	15	1.07 (0.53–2.17)	1.07 (0.62–1.83)
	≤50 cell/mm^3^	47	1.13 (0.76–1.67)	1.07 (0.75–1.53)	58	2.22 (1.24–3.96)	1.66 (1.07–2.64)
Discharge letter	Yes	115	Reference	Reference	NA	NA	
	No	23	1.82 (1.34–2.49)	1.47 (1.02–2.11)			

RR = relative risk, CI = confidence interval; ART = antiretroviral treatment.

Patients who died within 7 days of referral are not included in delayed or failed linkage to care.

Factors associated with increased risk of poor outcome of HIV referral in bivariate analysis were being ART-naïve at referral (RR 1.53, 95% CI 1.01–2.31 for delayed linkage to HIV care; RR 2.69, 95% CI 1.74–4.16 for failed linkage to HIV care), and referral to an inappropriate HIV clinic (RR 1.81, 95% CI 1.20–2.73 for delayed and RR 1.84, 95% CI 1.52–2.23 for failed linkage to HIV care) (Table 4). In addition, having a non-South African nationality (RR 1.27, 95% CI 1.01–1.59) and lack of HIV status and CD4 count information on the referral letter received at the TB clinic increased the risk of failure to link to HIV care (RR 1.86, 95% CI 1.49–2.34). There were no clear trends in association between CD4 cell count and delayed or failed linkage to HIV care. In multivariate analysis, being ART-naïve (RR1.29, 95% CI 1.10–1.52) and absence of HIV status and CD4 count on the referral letter (RR 1.23, 95% CI 1.10–1.38) independently increased the risk of failed linkage to HIV care.

**Table 4:** Factors associated with delayed or failed linkage to HIV care in 486 patients with HIV-associated TB referred from Helen Joseph hospital TB focal point to City of Johannesburg (COJ) clinic for HIV care and treatment.

		Delayed linkage to care* (>30 days) (n = 96)			Failed linkage to care* (n = 176#)		
		N	Crude RR (95% CI)	Adjusted RR (95% CI)	N	Crude RR (95% CI)	Adjusted RR (95% CI)
Sex	Female	54	Reference		90	Reference	
	Male	42	0.91 (0.66–1.27)		86	1.04 (0.84–1.28)	
Age	<36 years	47	Reference		89	Reference	
	≥36 years	49	1.04 (0.74–1.47)		87	0.98 (0.79–1.22)	
Nationality	South African	77	Reference		129	Reference	Reference
	Other	19	1.06 (0.71–1.60)		47	1.27 (1.01–1.59)	1.02 (0.88–1.19)
Education	≥Grade 8	76	Reference		136	Reference	Reference
	<Grade 8	15	1.13 (0.73–1.76)		28	1.13 (0.85–1.50)	1.03 (0.88–1.21)
	No education	5	1.41 (0.72–2.76)		11	1.40 (0.97–2.04)	0.95 (0.73–1.23)
Unemployed	No	45	Reference		78	Reference	
	Yes	51	1.03 (0.74–1.42)		95	1.06 (0.85–1.32)	
ART status	On ARV	21	Reference	Reference	17	Reference	Reference
	Not on ARV	75	1.53 (1.01–2.31)	1.56 (0.97–2.35)	159	2.69 (1.74–4.16)	1.29 (1.10–1.52)
CD4 count	>250 cell/mm^3^	16	Reference	Reference	32	Reference	Reference
	101–250 cell/mm^3^	34	1.05 (0.66–1.67)	1.13 (0.68–1.88)	42	0.82 (0.60–1.13)	0.97 (0.81–1.15)
	51–100 cell/mm^3^	18	0.64 (0.37–1.13)	0.73 (0.41–1.30)	22	0.52 (0.34–0.79)	0.86 (0.71–1.04)
	≤50 cell/mm^3^	16	0.77 (0.47–1.26)	0.72 (0.44–1.17)	32	0.90 (0.70–1.20)	0.93 (0.80–1.09)
Appropriate HIV referral*	Yes	84	Reference	Reference	140	Reference	Reference
	No	12	1.81 (1.20–2.73)	1.55 (0.92–2.59)	36	1.84 (1.52–2.23)	1.13 (0.93–1.35)
HIV status on letter	Yes	51	Reference	Reference	61	Reference	Reference
	No	45	1.32 (0.95–1.82)	1.28 (0.94–1.76)	115	1.86 (1.49–2.34)	1.23 (1.10–1.38)
Integration of referral	No	79	Reference	Reference	148	Reference	Reference
	Yes	16	0.71 (0.45–1.13)	0.79 (0.49–1.27)	28	0.73 (0.54–1.00)	0.95 (0.82–1.10)

CI = confidence interval; RR = relative risk;

# excludes patients who died within 30 days of referral;

* facility accredited for HIV treatment and care.

### Sensitivity analysis

Despite intensive investigations, 15.7% of patients could not be traced regarding linkage to TB care and 19.3% could not be traced to obtain information on HIV care. In the primary analysis, these patients were assumed not to have linked to care, which may have overestimated the rate of failed linkage to care. A sensitivity analysis in which all patients who could not be traced were classified as having linked to care, gave similar results compared to the main analysis. Being a non-South African (RR 1.30 95% CI 0.64–2.64), lack of formal education (RR 1.32 95% CI 0.61–2.88) and low CD4 count (≤50 vs. >250cells/mm^3^; RR 3.29 95% CI 0.42–25.97) resulted in a higher risk of failed linkage to TB care, but the association was no longer statistically significant, possibly due to the lower numbers of patients who failed to link to care in this analysis. Regarding linkage to HIV care, being ART-naïve at referral (RR 5.47 95% CI 2.06–14.49) and referral to an inappropriate HIV clinic (RR 1.93 95% CI 1.25–2.98) were associated with failure to link to HIV care in the sensitivity analysis; being a non-South African (RR 1.08 95% CI 0.69–1.70) and lack of HIV status and CD4 count on the referral letter received at the TB clinic (RR 0.94 95% CI 0.64–1.38) were no longer associated with failing linkage to HIV care in the sensitivity analysis.

## Discussion

We observed inadequate referral outcomes despite the implementation of a TB focal point infrastructure with a strong emphasis on integrated TB/HIV diagnosis. Almost one in four (23%) of patients failing to link to TB treatment at a primary care clinic, and 30% of those linking to care arrived after their 7 day supply of drugs expired. Linkage to HIV care was even more inadequate, with 38.3% of HIV-positive patients failing to present themselves to HIV care. Among HIV-infected TB patients, only 55.5% achieved linkage to both TB and HIV care, and one in six failed linkage to both TB and HIV care.

Our findings are in contrast to the observations by Edginton et al [16] who observed that the introduction of a TB care centre at another large hospital in Johannesburg, South Africa, improved patient referral with 93% of TB patients achieving successful referral to a clinic for continuation of TB treatment and nurses reporting that patients arrived “well-informed and motivated to continue treatment with referral documentation that were accurate and complete” [16].

Most socio-demographic characteristics, including age, gender and employment status, were not associated with poor outcomes of the referral process. Lack of formal education resulted in a two-fold risk of delayed and failed linkage to TB care, and being non-South African increased the risk by about 40%. As observed elsewhere, staff may not spend enough time on patient education and may not be able to communicate in the patient's native language [16]. In a Ugandan study, not being aware that TB is curable and not knowing the duration of anti-TB treatment was associated with poor linkage to TB treatment in patients with HIV-associated TB [23]. Foreign-born individuals and those who lack formal education may find it especially challenging to interpret information provided at referral and may be less empowered to seek continuation of care.

High rates of losses to care between HIV diagnosis and HIV care have been observed in many settings, highlighting the need for robust referral systems and a better understanding of risk factors [10]–[12], [24]–[27]. In our cohort of patients with HIV-associated TB, being ART-naive at time of referral was associated with a 30% increased risk of failed linkage to HIV care, and around a 40–50% increased risk for delayed linkage to HIV and TB care, although the latter was not statistically significant. In contrast to Basset et al [20], who found that patients with a CD4 cell count ≤100 cell/mm^3^ were twice as likely to be lost to care before ART initiation, CD4 count was not a risk factor for failure to link to HIV care in our population of HIV-infected TB patients. The increased risk of failure to link to HIV care among TB patients who are ART-naïve at time of referral highlights a lost opportunity for reducing early mortality among those with advanced immunosuppression [28]–[30] and suggest that initiation of ART during hospitalization, may not only improve survival but also reduce the likelihood of failure of linkage to care at primary care level.

The efforts towards an integrated TB/HIV approach at the hospital TBFP resulted in a 95.8% HIV testing rate and 84% of HIV-positive patients being evaluated by CD4 cell count. Unfortunately, the information on HIV status reached the primary care providers in only 23.7% of patients who linked to TB care, and the results of CD4 cell count were available at primary care level for only 18.8% of HIV-infected TB patients who linked to TB care. Failure to communicate HIV-related information to the health care worker, often because of confidentiality concerns, negatively impacts on patient care, as demonstrated by a 23% increased risk of failure to link to HIV care, and results in great inefficiency and high costs of duplicating efforts at different levels of the health care system. Our findings highlights the need to communicate HIV status and CD4 count at time of referral and suggest that national programs should consider revising the standard referral document if this does not contain designed entries for HIV-related information.

Integration of TB and HIV care at primary care level was poor, with 80% of patients with HIV-associated TB being referred to two separate clinics, one for TB and one for HIV care. The Pro-TEST study in urban Zambia demonstrated that delivery of integrated services, including a strong referral system reduces patient costs and is financially feasible and inexpensive [17].

Our study had several strengths, including the assessment of linkage to both TB and HIV care in a single cohort of patients, and extending data collection from the conventional review of registers or files at the referral clinic to review of an electronic HIV database, national death register, and telephone contact with patients who could not otherwise be traced [31]–[35]. The observation that 28% of patients who linked to care choose to go to a clinic other than the one they were referred to for continuation of TB treatment demonstrates the importance of this approach. Our study also had several limitations. First, even though the sample size was large, some estimates were imprecise due to small cell numbers in covariate strata. Second, despite intensive investigations, exact dates of arrival were missing in up to 9% of patients, 15.7% of patients could not be traced regarding linkage to TB care and 19.3% could not be traced to obtain information on HIV care. In the analysis, these patients were assumed not to have linked to care, which may have overestimated the rate of failed linkage to care. The results of a sensitivity analysis, in which these patients were classified as having linked to care, however suggested that potential misclassification did not substantially influence our conclusions.

In conclusion, the proportion of patients that failed to link to TB and HIV care was unacceptably high despite efforts to integrate TB and HIV care and streamline referral for patients diagnosed with TB at the hospital. The high rates of HIV co-infection and the high degree of immunosuppression in the population highlight the urgent need for strengthened linkages to and integration of care. Factors associated with linkage to care were predominantly health system related and could be improved through appropriate interventions. Efforts to improve linkage to care could include ART initiation during hospitalization to reduce the proportion of TB patients who are ART-naïve at time of referral; improving the quality and content of patient education and adapting referral information to the patient's level of education to overcome failure of linkage to care among those who lack formal education; discussing referral clinic options with the patient instead of simply assuming that the preferred clinic from the patient perspective is the one closest to their home; referring patients to a single clinic for integrated TB and HIV care; ensuring that ART-naïve patients are referred to a clinic that is accredited to initiate ART; and changing the standard reference referral letter to ensure that information on TB, HIV status and CD4 cell count is shared.
